# Preparedness and response against diseases with epidemic potential in the European Union: a qualitative case study of Middle East Respiratory Syndrome (MERS) and poliomyelitis in five member states

**DOI:** 10.1186/s12913-018-3326-0

**Published:** 2018-07-06

**Authors:** John Kinsman, John Angrén, Fredrik Elgh, Maria Furberg, Paola A. Mosquera, Laura Otero-García, René Snacken, Tarik Derrough, Paloma Carrillo Santisteve, Massimo Ciotti, Svetla Tsolova

**Affiliations:** 1 Department of Public Health and Clinical Medicine, Epidemiology and Global Health Unit, Umeå University, 901 87 Umeå, Sweden; 2 European CBRNE Centre, Umeå University, 901 85 Umeå, Sweden; 3 Department of Clinical Microbiology, Umeå University, 901 85 Umeå, Sweden; 4 Nursing Section, Faculty of Medicine, Autonoma de Madrid University, Madrid, Spain; 5 CIBER of Epidemiology and Public Health (CIBERESP), Madrid, Spain; 6 European Centre for Disease Prevention and Control, Granits väg 8, 171 65 Solna, Sweden

**Keywords:** Public health, Preparedness and response, MERS-coronavirus, Poliomyelitis, Cross-border, Inter-sectoral, Risk communication, Interoperability, European Union

## Abstract

**Background:**

EU Decision 1082/2013/EU on serious cross-border health threats provides a legal basis for collaboration between EU Member States, and between international and European level institutions on preparedness, prevention, and mitigation in the event of a public health emergency. The Decision provides a context for the present study, which aims to identify good practices and lessons learned in preparedness and response to Middle East Respiratory Syndrome (MERS) (in UK, Greece, and Spain) and poliomyelitis (in Poland and Cyprus).

**Methods:**

Based on a documentary review, followed by five week-long country visits involving a total of 61 interviews and group discussions with experts from both the health and non-health sectors, this qualitative case study has investigated six issues related to preparedness and response to MERS and poliomyelitis: national plans and overall preparedness capacity; training and exercises; risk communication; linking policy and implementation; interoperability between the health and non-health sectors; and cross-border collaboration.

**Results:**

Preparedness and response plans for MERS and poliomyelitis were in place in the participating countries, with a high level of technical expertise available to implement them. Nevertheless, formal evaluation of the responses to previous public health emergencies have sometimes been limited, so lessons learned may not be reflected in updated plans, thereby risking mistakes being repeated in future. The nature and extent of inter-sectoral collaboration varied according to the sectors involved, with those sectors that have traditionally had good collaboration (e.g. animal health and food safety), as well as those that have a financial incentive for controlling infectious diseases (e.g. agriculture, tourism, and air travel) seen as most likely to have integrated public health preparedness and response plans. Although the formal protocols for inter-sectoral collaboration were not always up to date, good personal relations were reported within the relevant professional networks, which could be brought into play in the event of a public health emergency. Cross-border collaboration was greatly facilitated if the neighbouring country was a fellow EU Member State.

**Conclusions:**

Infectious disease outbreaks remain as an ongoing threat. Efforts are required to ensure that core public health capacities for the full range of preparedness and response activities are sustained.

**Electronic supplementary material:**

The online version of this article (10.1186/s12913-018-3326-0) contains supplementary material, which is available to authorized users.

## Background

EU Decision 1082/2013/EU (October 2013) on serious cross-border health threats provides a legal basis for collaboration and information exchange between EU member states, and between international and European level institutions on preparedness, prevention, and mitigation in the event of a public health emergency [1]. This work entails a wide range of activities, including ensuring laboratory capacity for disease diagnosis; requiring hospitals to have plans in place to safely and effectively treat patients as well as to minimize the risk of nosocomial transmission; developing and maintaining strong surveillance systems to identify new cases; ensuring strong inter-sectoral collaboration, in particular between the relevant health and non-health sectors; and developing capacity for the provision of effective risk communication to the public and to health professionals [2] [3] [4] [5] [6].

Among the health threats that are considered important within the context of EU Decision 1082 – insofar as an outbreak in one EU country potentially represents a threat to all the others – are poliomyelitis and respiratory diseases such as Middle East Respiratory Syndrome (MERS) [7]. The importation and spread of MERS or a reintroduction of poliomyelitis into the EU could have significant social, economic and political ramifications. In this sense, the legal framework provided by EU Decision 1082 offers an effective basis for discussions about European preparedness and response.

MERS and poliomyelitis are very different diseases, both clinically and epidemiologically. MERS is a respiratory infection, first identified in 2012 [8] and now seen as one of the top emerging pathogens with potential for causing a severe outbreak [9]. It appears that the ultimate source of most MERS outbreaks is camels who infect humans, with most human cases – whether autochthonous or imported – caused by secondary or tertiary nosocomial transmission within the healthcare sector [10] via an infected person’s respiratory secretions [11]. As of April 4, 2017 there had been 1936 cases reported by health authorities worldwide, of whom 690 (36%) had died [12]; while within the EU there have been 15 cases, with 7 (47%) deaths [13] [14]. MERS has not been declared by WHO to be a Public Health Emergency of International Concern (PHEIC), but the Committee responsible for this decision has nonetheless stated that “*the progress [made to date against MERS] is not yet sufficient to control this threat and until this is achieved, individual countries and the global community will remain at significant risk for further outbreaks*” [15].

Where human-to-human infection does occur (usually to members of the same household of the index case, other patients, or health care workers [16]), the mean number of secondary cases generated by a typical infectious individual, called R_0_, has been calculated as generally being < 1 [17]. In other words, the outbreak would die out by itself. However, a MERS outbreak in South Korea in 2015 – introduced by a single imported case from Saudi Arabia – illustrated the dramatic effect the disease can have on the local or national healthcare systems. This outbreak, involving 185 cases, was driven primarily by delayed diagnosis of a few index patients, with the result that the reproductive number (R_0_) greatly exceeded 1 in the early stages of the outbreak [18] [17]. It is not known whether these individuals became super-spreaders for clinical, virological, environmental, or social reasons, and it is precisely this uncertainty which highlights the importance of good preparedness against a potential upsurge in MERS cases in Europe.

By contrast to MERS, poliomyelitis is a vaccine preventable disease, spread predominantly by the faecal–oral route. Significant resources have been dedicated to bring about the global eradication of polio, but on 28 May 2014, WHO declared the ongoing international spread of poliomyelitis to be a Public Health Emergency of International Concern (PHEIC) [19]. The WHO European Region has been officially polio-free since 2002 [20], but this status has been challenged several times in recent years by events in nearby countries. In 2013–2014, wild poliovirus was detected in sewage samples in Israel, and cases of paralytic poliomyelitis were confirmed in Syria at a time when several EU countries were receiving refugees from Syria fleeing the political unrest [21]. In addition, two cases of circulating vaccine-derived poliovirus (cVDPV) were confirmed in Ukraine in July 2015 [22].

Continued vigilance against the two diseases in Europe is, therefore, essential. Given this, the European Centre for Disease Prevention and Control (ECDC) has initiated a series of case studies to review the public health preparedness status of the health and other relevant sectors in three EU Member States with respect to MERS (the UK, Greece, and Spain, which to date have had 4, 1, and 0 cases respectively) and two EU Member States with respect to poliomyelitis (Poland and Cyprus). Poland borders the region of Ukraine where the outbreak of cVDPV was identified in 2015; while the migrant crisis may place Cyprus at risk of poliomyelitis importation. The European Regional Certification Commission for Poliomyelitis Eradication’s (RCC) 2015 report concluded that both Poland and Cyprus were considered to be at ‘intermediate risk’ of subsequent transmission of poliovirus in the event of an imported case [23].

In addition to its being a serious emerging infectious disease in its own right, MERS was taken as a case study that could provide insights into preparedness planning for respiratory diseases including influenza and pandemic influenza, as well as into the links between human and animal health. Poliomyelitis was included as a case to review preparedness planning for vaccine preventable diseases targeted for elimination by WHO with the potential for serious outbreak impact, along with related environmental and containment issues. The selection of countries was based on (a) actual cases or the potential for cases to emerge due to proximity of the health threat, and (b) an expression of interest to participate from the country.

This is the background for the present paper, which aims to identify good practices, lessons learned, and gaps in preparedness and response to MERS and to poliomyelitis respectively in the five countries. This aim is in line with EU Decision 1082/2013/EU [1], which provides the legal framework for addressing cross border health threats, including analysis of preparedness planning, interoperability between health and non-health sectors, business continuity, and cross border collaboration. The study also aimed to provide insights into the ways in which the capacities and capabilities for these domains (concerning, for example, training, simulation exercises, risk communication, and challenges in the implementation process) have been considered by the key stakeholders. Based on our findings, we propose approaches for strengthening public health emergency preparedness plans for the five participating countries and for other EU Member States.

## Methods

This study used a qualitative case study methodology [24], which included (i) a documentary review, based on published and unpublished materials; and (ii) a series of face-to-face, open-ended, semi-structured interviews and focus groups with experts from both the health and non-health sectors who either played some role in responding to the MERS case/s in their country, or who are engaged in preparedness and response activities against polio.

In order to minimise bias in the documentary database, the material collected came from two independent sources: internet databases, which were searched by our research team, and which produced predominantly English-language material; and documents provided to us by each country’s ECDC National Focal Point (NFP) for Preparedness and Response,^1^ who was our key country contact.

The internet searches involved databases such as Google Scholar and PubMed, using search terms including the name of the country, ‘preparedness plan’, ‘MERS’, ‘influenza’, ‘polio’, ‘standard operative procedure’, etc. Material provided by the ECDC NFPs included (i) Policies that may be related to the EU Decision on serious cross-border public threats; (ii) Relevant reports and findings from previous EU-supported studies; (iii) Standard Operating Procedures, contingency plans and guidelines in the event of a pandemic respiratory disease threat; (iv) Lessons learned from any simulation or training exercises that may have been conducted. Some of this was available only in the local language, so it was translated into English using online software, thus enabling us to develop a good sense of the key points made.

Analysis of the documents was conducted on a thematic basis (i.e. including preparedness plans, inter-sectoral collaboration, risk communication etc.), with the major points on each theme extracted and reviewed carefully by the research team prior to the field visit. This process served to provide an understanding of the specific country context, as well as to inform the questions that we asked during the interviews.

For the interviews and focus groups, the ECDC NFP and his/her institution also took responsibility for organizing an intensive week of meetings with representatives from key agencies and sectors. The criteria for selection reflected the specifics of the respective case studies: for MERS, as a respiratory infection, experts were interviewed from the national public health authorities, the Ministry of Health, ambulance services, laboratory specialists, infectious disease doctors; and from non-human health sectors such as travel, transport, health journalism, and animal health. For polio, the National Certification Committee for Polio was also represented, along with non-health sectors such as border control, the Interior Ministry, and non-governmental organizations such as the Red Cross. Once a provisional list of relevant experts had been agreed, contact was made with them by the country counterparts to arrange for an interview.

The interviews and focus groups for MERS were conducted in the UK (England), Greece, and Spain during September–October 2014; while those for poliomyelitis were conducted in Poland and Cyprus during November 2015. A total of 35 and 26 interview sessions were held for MERS and poliomyelitis respectively; of these, 36 were with people from the health sector and 25 were with people from non-health sectors. See Table 1 for a summary, and Additional file 1 for details of the interviewees’ affiliations.

**Table 1:** Number of interview sessions for the two investigated diseases, conducted by sector, in each of the five participating countries

	Disease	Health Sector interview sessions	Non-Health Sector interview sessions	Total number of interview sessions
UK (England)	MERS	5	6	11
Greece	MERS	6	6	12
Spain	MERS	7	5	12
Poland	Polio	9	4	13
Cyprus	Polio	9	4	13
TOTAL		36	25	61

Individual interviews were conducted whenever possible, but in some cases, focus groups of different sizes were organised in order to involve more participants in the discussion, thereby enabling us to record opinions from different stakeholders at the same time, and to hear a wider range of perspectives. The interview sessions in Greece, England and Spain included from one to three respondents each; the numbers were similar for most sessions in Cyprus and Poland, but some of the sessions involved group discussions with up to 8 and 20 respondents respectively. We had requested for the focus groups to include no more than 10 people, but local organisers and the willingness of experts to participate sometimes added to that number. Similar questions were asked in the individual interview as in the group interviews. Material from the interviews and the focus groups was combined for the analysis.

For the interviews and focus groups, we developed pools of questions for both MERS and polio, in cooperation with the ECDC NFPs, which aimed at reflecting the objectives of the study concerning planning and preparedness policies and practices, risk communication, training and exercises, vaccination (for polio), surveillance, and issues to do with inter-sectoral and cross-border coordination. One over-riding sub-set of the questions was asked to all interviewees, regardless of their particular position; another sub-set of questions concerned issues specific to the interviewee’s particular sector or position; and a final sub-set of questions was used only if time permitted. Interviewees were also free to discuss other, additional issues that they considered to be important, but which were not included in our pre-determined list. Through this process, we received responses to each question from a range of different people in different positions, thereby facilitating triangulation of the data in each country. We do not claim to have achieved data saturation in all the issues covered, but, because of the range and expertise of the people we interviewed in each country, we are confident that all the major relevant points were raised.

Oral and, where feasible, written informed consent was sought and obtained from participants after explaining the objectives of the study. Interviews were conducted face-to-face, either at the interviewees’ office or at the ECDC NFP’s home institution; and interviewees took part purely in their professional capacity.

Most of the interview sessions lasted around 60 min. All except for four of the interviews in the UK, Greece, Poland and Cyprus were conducted in English; professional interpreters assisted in these cases. The interviews in Spain were conducted in Spanish by native speakers. All five country visits were focused primarily on the national level, but in Poland, a 24-h visit was made to Rzeszów in the south east of the country, 300 km from Warsaw and near to the Ukraine border; and in Cyprus, the team spent half a day at an asylum and refugee centre at Kofinou, 50 km from Nicosia.

The interviews were conducted by researchers based at Umeå University, Sweden, which was contracted by ECDC to do this work. A senior researcher conducted the interviews in each country, supported by a junior researcher who took notes. Most of the MERS interviews were digitally recorded as an additional back-up, but, based on our experience from the MERS phase of this work, we considered it unnecessary to record the poliomyelitis interviews, so we relied exclusively on the notes instead.

The interviewers were accompanied to many of the interviews by the ECDC NFP or his/her representative, which greatly facilitated the introductions and the presentation of the study objectives. The interviewees had received the questions in advance of our visit, so they were able to review the topics and prepare their responses as they saw fit.

After each interview, the investigators exchanged views on what had been discussed, and agreed on some of the key points raised. Notes were finalized at the end of each working day, and collectively these constituted the dataset used in the production of five country-specific reports.

A debriefing meeting was held after the last interview on the last day of each country visit, including the two interviewers, the ECDC NFP, and representatives from the national public health authorities, the Ministries of Health, Internal Affairs and other invited representatives, and colleagues from ECDC in Stockholm (via teleconference link). This discussion constituted the first formal review of the week’s work, and as such proved to be a valuable moment for reflection and validation of the initial analysis.

When the field work week was over, the data were subjected to thematic analysis, based on a number of pre-determined themes (e.g. preparedness planning, risk communication, training etc., as delineated above), but a few other themes also emerged inductively from the data during analysis. These analyses provided the basis for country-specific reports, each of which were then sent to the respective ECDC NFP for review and an opportunity to correct, clarify and otherwise comment on the conclusions drawn. In turn, the five (unpublished) country reports formed the basis for the present article.

## Results

The findings from the country visits are presented collectively below, in six broad themes, and with commentary on both MERS and polio. Where possible, countries are anonymized in this discussion. Similarly, we have not used quotations in the text, as agreed with the National Focal Points in the participating countries, in order to preserve confidentiality.

### National plans and overall preparedness and capacity

The overall perception of our interviewees in all five participating countries was that the level of preparedness for MERS and poliomyelitis respectively was high. Clear legal frameworks exist which indicate specific roles and responsibilities, and the key actors in each country appeared to be well informed about these. Networks with appropriate resources and diagnostic capacity for the two diseases also exist in all the countries. However, although these are well-integrated and properly functioning formal systems, informal personal networks and contacts both within the health sector and between the health and relevant non-health sectors were widely considered as being key to the effectiveness of process and practice in preparedness and response.

In countries where MERS preparedness was analysed, pandemic influenza preparedness plans exist in each of the UK, Greece, and Spain, and, as a respiratory infection, these were seen as being of at least some relevance to MERS (see https://ecdc.europa.eu/en/seasonal-influenza/preparedness/influenza-pandemic-preparedness-plans). However, while these plans are in the public domain and are therefore easily accessible, they have not been updated for several years, which points to potential gaps in preparedness. Operationally, we were informed that much knowledge and experience has been gained through various global public health events since the beginning of the century, such as Severe Acute Respiratory Syndrome (SARS, 2003), the influenza A(H1N1)pdm09 pandemic (2009), and Ebola (2014–2016); and many of the practices and lessons learned from these threats have been sufficiently generic to prepare frontline health workers and the relevant authorities for MERS. One of the major lessons learned from these events has been the necessity to develop systems for increasing health care capacity in the event of a pandemic or a major outbreak. We were informed that these systems include plans to utilise the private sector if the public sector is overwhelmed by, for example, MERS cases; and also the reorganisation of hospital ward structures such that an entire ward could be given over to patients with MERS, thereby minimising the danger of cross-infection between patients in different wards.

In relation to polio, all EU countries are obliged by the WHO’s Regional Certification Commission for Poliomyelitis Eradication (RCC) to ensure that they have poliomyelitis preparedness plans in place, including access to vaccine in case of an outbreak or plans on how to get some; and, through their obligatory annual reports to the RCC, Cyprus and Poland have both shown that they comply with this [23]. Overall poliomyelitis vaccination rates are high in both Cyprus and Poland (97 and 92%, respectively for the three doses, according to 2015 data [25]), but both countries nonetheless have relatively small but still significant numbers of refugees and populations that are hard to reach in vaccination campaigns (e.g. Roma people) and which are therefore vulnerable in case of a poliomyelitis virus outbreak. Under such circumstances it is important to have well-functioning surveillance systems in place in order to identify potential poliomyelitis cases. Acute Flaccid Paralysis (AFP) surveillance is regarded as the gold standard for detecting cases of poliomyelitis [26]; it involves finding and reporting children with AFP; transporting stool samples for analysis; isolating and identifying poliovirus in the laboratory; and mapping the virus to determine the origin of the virus strain. According to WHO guidelines, environmental surveillance can be justified in some specific situations in addition to AFP surveillance [27]. Environmental surveillance involves testing sewage or other environmental samples for the presence of poliovirus [28]. Both countries have AFP surveillance systems in place. At the time of the study, environmental surveillance systems have not been introduced, however, but the requisite technical and scientific capacity does exist, should a decision be taken in the future to bring them in.

The financial crisis and subsequent austerity measures that have affected many European countries since 2009 have had a significant adverse effect on preparedness and response capacity in some countries. We were informed of budget cuts that have adversely affected recruitment of new staff as well as opportunities for trainings and other exercises. However, we were informed that emergency funding is, or would be provided in the event of a public health emergency, either directly from governmental structures tasked with dealing with crises (i.e. civil protection or Prime Minister’s crisis centres), or, if such arrangements exist within the legal framework of a particular country, from the Ministry of Health.

### Training and exercises

Training and exercises (including simulation exercises (SIMEX)) are recognised as key components of any efforts to sustain public health preparedness capacity, both through their ability to identify weaknesses in the systems and because they provide a basis for developing networks of professionals that could be called upon during a public health emergency [29]. Training and exercises can be conducted within a single country, ideally including both the health and all the relevant non-health-related sectors; or as part of a multi-country process involving neighbouring countries, including other EU Member States and/or non-EU neighbouring countries. Exercises could also provide an external impetus that demands a review of national plans, which may otherwise be seen as a low priority activity. Further, they provide an opportunity to retain staff capacities and institutional memories, including whatever lessons may have been learned from recent public health emergencies, as well as preparedness legacies from, for example, the Olympic Games (held in Greece in 2004, and the UK in 2012).

However, in at least one of the three countries we visited in relation to MERS, cuts in funding had been made for general preparedness and training activities, and national exercises had been cancelled. This significantly reduced the opportunities to enhance preparedness and response measures in the event of a serious public health threat to the country. In another country, the national preparedness plan called for exercises to ensure that business continuity arrangements are in place for the emergency services; and training for personnel whose work will oblige them to wear respirators. However, no details were given regarding how often or how extensive these trainings should be. It was suggested by our interviewees that executing table-top preparedness exercises may prove to be a less expensive, and therefore more feasible alternative than conducting full-scale simulation exercises. Where possible, these could be organised by national authorities, complemented as appropriate with input from international organisations.

The absence of poliomyelitis in Poland and Cyprus since 1984 and 1995, respectively [30] [31] has diminished practical, hands-on experience of dealing with the disease. It has also diminished the perceived imperative for poliomyelitis preparedness, and as such, there have been no poliomyelitis preparedness exercises conducted in either country in recent years. Since a rapid and effective initial response is essential for controlling a poliomyelitis outbreak [32], this could lead to delays and a compromised response if one was to occur. That said, some interviewees did recommend that support could be provided from, for example, ECDC for a simulation exercise or training, either at national or regional level and involving all the relevant sectors, with a particular focus on risk assessment and incident analysis, alongside a review and discussion of existing outbreak response guidelines for polio.

### Risk communication

In each of the three countries we visited for MERS, media and communications experts in the respective national-level public health institutions are mandated to lead risk communication efforts for the public and for health workers during a respiratory disease epidemic or pandemic. These included the Hellenic Centre for Disease Control and Prevention in Greece, known by its Greek acronym as KEELPNO; Public Health England (PHE) in the UK; and the Coordination System for Health Alerts and Emergencies (SICAS) in Spain. One of these institutions had produced health-promoting materials that were relevant for a pandemic situation, and stored them on hidden webpages that could instantly be made public should the need arise. This is a useful model that could be applied elsewhere. Since a reportedly significant potential challenge during a pandemic concerned communicating with migrant, hard-to-reach, and non-native-speaking populations, it was pointed out that all relevant health-promoting materials should be translated into languages used by such people. Without this, they may not know how to take the necessary steps to protect themselves.

For polio, we were told that one of the most important challenges facing risk communicators who work with vaccine preventable diseases (VPDs), and specifically those who work with polio, is the fact that the public does not feel especially at risk from these diseases. With Cyprus and Poland having been polio-free for over 20 and 30 years, respectively, memories of the disease have largely faded and a majority of people are simply unaware of its potential severity. Thus there is reduced public acceptance of vaccination alongside an impression that the benefits of some vaccines may, within the current epidemiological context, only marginally outweigh their potential risks [33]. This, in combination with the fact that there could be issues with trust in some public authorities in some of the countries visited means that providing vaccine-promoting information that is trusted, believed, and acted upon requires a carefully developed strategy. Solutions suggested to us included (i) the public health authorities making systematic efforts to understand vaccine hesitancy where it exists, and responding proactively to people’s concerns using appropriate information that is already available from, for example, ECDC, WHO-Euro, and the Global Poliomyelitis Eradication Initiative; (ii) conducting trainings about vaccination for journalists, who might need to be better informed about the topic so that they do not inadvertently perpetuate myths and misperceptions about vaccination; and (iii) enhanced use of social media by the public health authorities.

An important finding from several of the countries we visited was that evaluation of the risk communication strategies operated by the public health authorities is often limited or non-existent. Thus the effectiveness of the strategies is unknown, as is the extent to which the messages could be misunderstood or misinterpreted.

### From national policy to local level implementation

The relationship between the national and local levels is critical for ensuring continuity between policy and implementation, but in each of the three countries that we visited for MERS, challenges of different sorts were identified regarding the implementation of national policy at local level. These challenges arose either as a result of reportedly insufficient financial or human resources at local level, or because of particular structures or policy divisions between national, regional and local level. For example, while the decentralisation in one of the three countries was seen as a strength – as the local structures themselves developed the operational plans and as such these were ‘owned’ by the people who would implement them – we were also told that local authorities in that same country had differing financial capacity to implement activities above the nationally required minimum level, which could result in sub-optimal coordination of the national pandemic response, with different kinds and quality of activities in different regions.

Countries may also experience significant shortages of qualified personnel in some peripheral areas, with the result that the preparedness and response infrastructure in those places could potentially be sub-optimal. However, it was suggested that at the local level there may be areas with better inter-sectoral collaboration and coordination than many major urban centres, simply because people in the different sectors often know each other personally. In that sense, the limitations in one issue may be offset to some extent by the advantages in another.

In the case of poliomyelitis preparedness, both the countries that we visited appeared to have structures in place to ensure a coordinated and effective flow between national policy and local level implementation. As with many small countries, the Cypriot system includes some minor local level administrative functions, but since the country is so small, a large proportion of the national level administrative, legislative and organizational work is effectively also local. Operationally, this results in a system that does not give much room for decision making power at the local level, but it also means that policies and directives tend to be easily followed and implemented.

Poland, as a much larger country, operates on a largely decentralized basis. District level authorities have authority to enforce public health regulations, including quarantine if necessary; but the Ministry of Health in Warsaw provides guidelines for the lower administrative levels in order to ensure uniformity in planning and implementation. We were not informed of any significant weaknesses in this system.

### Interoperability between the health and non-health sectors

One of the most important non-health-related sectors of relevance for MERS is, we were told, air travel. If MERS spread widely in a country, then Civil Protection agencies would be engaged, as would Border Control. In addition, working with journalists would be key to ensuring an effective response (as suggested under *Risk Communication* above). Collectively, these sectors represent a wide array of different actors, and ensuring interoperability between them and the health sector could represent a challenge. For example, interoperability with Border Control was reportedly poor in one of the countries we visited, with personnel said to be ill-trained regarding what to do if presented with someone presenting with respiratory distress.

By contrast, those sectors that have a clear financial incentive for controlling infectious diseases – including agriculture/animal health, tourism, and air travel – were seen as more likely to have in place public health preparedness and response plans that were interoperable with those of the health sector. Within the agricultural sector, for example, while there are very few camels in Europe, recent evidence points to the possibility that pigs are susceptible to the MERS virus [34]. The historically strong collaboration between the animal and human health sectors that exists in many countries provides a good basis for addressing the potential risks arising from this finding. Such collaboration can be seen within the context of the ‘One Health’ approach, which “recognizes that the health of humans, animals and ecosystems are interconnected, [and which] involves applying a coordinated, collaborative, multidisciplinary and cross-sectoral approach to address potential or existing risks that originate at the animal-human-ecosystems interface” [35].

Safety is of course also a primary concern for airlines, as their very survival depends on ensuring safe travel. Consequently, systematic thinking about safety has been fully integrated into all aspects of this sector. At one airport we visited, we were told of a generic pandemic preparedness plan, which includes updated lists of key contacts for all the key institutions involved (including the national public health agency, the ambulance service, and major hospitals), and which lists clear standard operating procedures for different eventualities. If an aircraft arrived with a passenger on board who appeared to be carrying a serious infectious disease, for example, air traffic controllers would be obliged to obtain as much information as possible from the pilot in advance of their arrival, and this would be forwarded to the airport authorities who would then contact the national public health agency. Once landed, the aircraft must follow clear rules about where it is to be parked, and how the disembarking passengers are to be processed.

With regard to polio, we were told that the relevant sectors in both countries included Border Control and the Interior Ministry for the border regions, and, more widely, those responsible for managing migration and refugees. The formal procedures for inter-sectoral collaboration are reportedly not always as clearly delineated as they are for the sector-specific work, in part due to what was described as the ‘vertical modes of communication’ in the respective sectors. Overall, the success of inter-sectoral collaboration in poliomyelitis preparedness and response relies more on personal contacts between the key actors than on formal protocols, at least in part because there have been no cases for many years, and therefore familiarity with these protocols is limited. A local level example in one country was described to us as having very well organized inter-sectoral collaboration on poliomyelitis preparedness, involving the health authorities, the border guards, and the Ministry of the Interior, but this was because the key actors in this region know each another personally, and they meet through their work on a regular basis. However, effective and sustainable inter-sectoral collaboration on this basis is vulnerable to individual personnel moving from one official position to another, or otherwise being unavailable in a crisis situation.

### Cross-border collaboration

We found few significant disease-specific issues in relation to cross-border collaboration. Rather, the nature and extent of cross-border collaboration in addressing health threats appears to be determined primarily by the larger political context, as well as by the formal and informal relationships that may have developed between individuals and institutions on both sides of the border or borders in question.

For example, our findings suggest that a key determining factor relating to cross-border collaboration is whether or not the neighbour in question is a fellow member of the EU. As a general rule, collaboration and information exchange is greatly facilitated if it takes place between two EU Member States as opposed to between an EU Member State and a non-EU Member State. For example, the Early Warning and Response System (EWRS) was cited by interviewees in several countries as an invaluable instrument for keeping abreast of infectious disease developments in fellow-EU countries. Operated by ECDC, EWRS is an internet-based system for sharing information about health alerts between EU Member States. It includes an option for sending a copy of any message to WHO if the information might be of wider concern, for example in the context of the International Health Regulations. EWRS does not operate outside the EU.

In one country that we visited with a non-EU Member State neighbour, two distinct perspectives of cross-border relations emerged in the interviews: the national level perspective, and the local level perspective. At national level, the flow of health information between the two capital cities was reportedly very limited, in spite of friendly relations between the countries, with most information about the other country reaching our hosts via WHO and ECDC. This was due to quite different organisational cultures between the two countries, which complicated communications. Consequently, the International Health Regulations provided the only real means for our hosts to learn about events in the other country.

At local level, by contrast, there was a very good exchange of information between the respective border control authorities, based on a longstanding bilateral legal agreement. Each border post between the two countries had personnel whose jobs specifically included communicating with their counterparts across the border. Further, border guard commanders in both countries were obliged to immediately notify their counterparts across the border in the event of any sudden and unexpected illness or disease that was identified in the area under their jurisdiction. However, much of this information stayed and was acted upon at the local level, and – because it was operational as opposed to strategic – it was not sent to national level.

In spite of the stated advantages of working with fellow EU Member States, one challenge was mentioned that arises from the EU’s Schengen Agreement. At least up until the current refugee crisis, there has been free movement between all signatory countries, with no form of border control. This means that once people have entered into the Schengen Area – even if they have originated from a high-risk country for a particular infectious disease – there is no realistic way of systematically following up on their health status or ensuring that they receive health care as necessary. As MERS cases at early stages and poliomyelitis infections are usually asymptomatic, enhanced epidemiological surveillance and early detection therefore remain the best preventive measures. For poliomyelitis, WHO recommendations for travellers to and from countries where the virus is circulating to be vaccinated should be followed [36].

Participation in European and other international disease surveillance networks and associated research projects was recognised as playing an important role in maintaining high levels of cross-border preparedness. Similarly, simulation exercises involving several countries were described to us as being invaluable opportunities for identifying weaknesses in preparedness systems, and for creating the basis for strong, cross-border professional networks that could prove critical in tackling cross-border health threats. The EpiSouth project was cited as an example of this [37], which included both EU and non-EU Member States from the entire Mediterranean region. Such exercises are expensive, however, and EU support for more such multi-country simulation exercises, possibly also including non-EU countries, was suggested as a potentially good investment.

## Discussion

This qualitative study has investigated the public health preparedness and response efforts of five EU member States, with specific reference to MERS-Coronavirus and polio. Aspects of the work have previously been published as ECDC Technical Reports [38] [39], but we are not aware of any other comparable multi-country research into public health preparedness and response in Europe. As such, the findings provide important insights into some of the current preparedness and response processes in the region.

The overall finding from this work is that there is a high level of technical expertise available to implement existing preparedness and response plans for MERS and polio, in the event that this became necessary. The fact that the formal protocols were not always up to date was to some extent offset by what appeared to be good personal relations within the relevant professional networks, which could be brought into play in the event of a public health emergency. It was also clear to us that the individuals and institutions concerned well understood the relevant legal frameworks as well as the operational procedures that would have to be followed if there was a MERS outbreak or a polio event in their country.

The most significant preparedness challenges that we identified were the result of pressures on public budgets and associated restructuring and reorganization of public health systems, which brought about consequences ranging from limiting the opportunities for training and exercises; making it more difficult to recruit and retain experienced, well trained staff; and for systematically identifying good practices and lessons learned that could be incorporated into protocols. Although our study design did not permit significant investigations into local-level variations in preparedness [40], budgetary challenges have been reported before in other contexts at both national and local levels [41] [42] [43], so it is likely that this challenge is also reflected at local level in at least some of the participating countries. An overriding conclusion, therefore, is that countries must ensure that sustainable human resource and funding capacity for public health preparedness and response activities is secured at all administrative levels, based on systematic risk assessment and risk ranking exercises.

By conducting this research on two different diseases, we have observed areas in preparedness and response where all-hazard, generic plans may be suitable [44] [45], and other areas where more disease-specific plans are needed. For example, in relation to cross-border collaboration, it was seen as more important to have protocols that ensure open communications between the authorities on both sides of the border than to have a disease-specific plan. By contrast, the content of risk communication messages clearly needs to be disease-specific in order to inform the public how best to respond to a given threat. The discussion below outlines some of the issues, both generic and disease-specific, that have emerged through this study.

With regard to cross-border communications, the International Health Regulations (IHR) and Early Warning and Response System (EWRS) were cited as essential tools for staying abreast of events in other countries, and ECDC also manages and facilitates disease-specific communication platforms that have proven useful for a variety of disease groups, such as food-and waterborne disease and vaccine-preventable disease. Such a platform does not, however, exist for respiratory diseases. Conducting cross-border simulation exercises during ‘peacetime’ was therefore seen as one way of developing the requisite networks and contacts to ensure good levels of preparedness and response for such diseases [46]. Since exercises can be costly, a legal framework is found in Article 4 of EU Decision 1082 that could be used to leverage funds to support them.

As with cross-border communications, the development of good inter-sectoral coordination and collaboration should also be prioritised in public health preparedness activities [41]. We saw efforts towards this in all the five countries we visited, though it occurred to different degrees in each of the five countries and between the different sectors therein: formal procedures or protocols aimed at ensuring good inter-sectoral collaboration [47] are not always in place. Where such protocols do not exist, they should be drafted collectively by the relevant authorities and circulated to those who will implement them; and where they do exist, they should be regularly reviewed and updated as appropriate. An additional strategy that has reportedly improved inter-sectoral coordination in preparedness initiatives is the ‘meta-leadership summit’, which provides a venue for leaders of different organizations and sectors involved in decision making during a major emergency to learn the concepts and practice of multi-sectoral collaboration and resource-sharing [48].

Overall, high technical capacity and preparedness levels were observed for polio. The absence of environmental surveillance in both Cyprus and Poland, reportedly due to financial or policy constraints, remained a concern for some of our interviewees. They pointed out that relatively modest costs may be incurred by an environmental surveillance programme over the short-to-medium term, but such a programme could nonetheless be seen as a potentially cost-saving exercise over the longer term, since it could identify a poliomyelitis outbreak early on, thus triggering a rapid response [32] which would significantly reduce its spread, impact, and cost.

One systemic weakness identified in our study was that, in several of the countries visited, relatively little of the risk communication work conducted during previous public health events has been subjected to formal evaluation – a problem that has previously been reported in other areas in public health [49]. Consequently, there is limited documentation about the lessons that have been learned, which means that effective risk communication during future public health threats may have to rely on the relevant institutions having retained those individuals who have previously been engaged in key positions. This cannot be taken for granted, especially in countries where austerity measures have significantly cut public health budgets, and consequently there is the risk of mistakes from the past being repeated, while good practices are forgotten [50]. One way to address this gap would be for Member States to receive training and support from agencies such as ECDC to develop their evaluation capacity. This could be beneficial both for risk communication specifically, but also more broadly in relation to other aspects of public health emergency response.

### Study strengths and weaknesses

Some reflections on our methodological approach are due here. Perhaps the major advantage of our overall strategy – the use of foreign, or otherwise external social scientists to conduct open-ended interviews with in-country experts – was that it provided the opportunity for the latter to reflect on their work in a way that can offer a fresh perspective on what may often seem self-evident to them as ‘insiders’ [51].

Further, the multi-sectoral perspective that we took provided us with a much wider lens through which to view the different countries’ preparedness activities and capacities than would have been gained had we focused only on the health sector. From the participating countries’ points of view, our meetings with stakeholders from non-health-related sectors brought people out of their sectoral ‘silos’, and, in this sense, the research process itself contributed to awareness raising about public health preparedness, and to facilitating contacts between the different stakeholders and sectors.

In spite of these clear advantages, there were also practical challenges inherent in our methodology, not least of which were the time and effort required by the national counterparts to organise the interviews and the schedules for our visits. Language issues also presented challenges: all the interviews in Spain were conducted by native Spanish speakers, but not all interviewees in Poland, Cyprus or Greece were comfortable or able to communicate effectively in English, which meant that on a few occasions we had to bring in professional translators. This had cost implications, and, although the translators were invariably excellent, some nuances may have been lost. In addition, the research team was not able to understand all the documentation sent to us by the ECDC NFPs in these countries, which meant we had to use translation software. Again, although this facilitated our understanding the main points, some of the finer details may have been lost.

Finally, this was an exploratory study that covered a broad range of topics, based on a limited number of key informants per country. A number of areas were identified that would benefit from more detailed investigation in future. For example, we did not review the participating countries’ health systems structures or organisations in relation to their preparedness and response capacity, even if these are clearly important for ensuring good overall coordination at regional and national level. In addition, we did not manage to gather rich material on business continuity, risk assessment, or on the interoperability of plans between different sectors in the event of a public health emergency. We believe that our overall methodological approach could be used as the basis for designing a more comprehensive study, including on these specific topics, aimed at further improving our understanding of preparedness planning and implementation processes.

## Conclusions

This qualitative study has investigated preparedness and response measures to MERS and poliomyelitis in five different EU Member States. Plans and capacities do exist in all the countries, and important lessons have been learned and institutionalised based on experiences from recent public health emergencies, such as SARS (2003), the H1N1 pandemic (2009), and Ebola (2014–2016). There have also been substantial legacy benefits from recent mass gatherings such as the Olympics, which were held in Greece in 2004, and the UK in 2012.

However, the innovations, experience, and training from these events are vulnerable to political and policy prioritisation moving away from ensuring sustainable core public health capacities in some of the participating countries. Further, formal evaluations of major public health events are not systematically conducted in all the countries, whereby lessons learned may be documented for use in future emergencies. Since much of the strength in the systems that we have observed is based on good personal connections between key professionals who work together, these systems are vulnerable to the possibility that key individuals are incapacitated or otherwise unavailable during an emergency. This applies in particular in relation to inter-sectoral and cross-border coordination, which, within the context of EU Decision 1082, are core components of public health preparedness in Europe today. Conducting evaluations, documenting the findings, and disseminating these widely through regular training of all relevant personnel are therefore essential activities for ensuring the resilience of European public health preparedness systems.

## Additional file

Additional file 1: Sectors and affiliations of the interviewees in the five participating countries. (PDF 436 kb)

## Abbreviations

AFP: Acute Flaccid Paralysis
cVDPV: Circulating Vaccine-Derived Poliovirus
EU: European Union
EWRS: Early Warning and Response System
GPEI: Global Polio Eradication Initiative
IHR: International Health Regulations
MERS: Middle East Respiratory Syndrome
NFP: National Focal Point
PHEIC: Public Health Emergency of International Concern
RCC: Regional Certification Commission for Poliomyelitis Eradication
SARS: Severe Acute Respiratory Syndrome
SIMEX: Simulation Exercise
VDPV: Vaccine-derived poliovirus
VPD: Vaccine Preventable Disease
WHO: World Health Organization

## Footnotes

1 Each EU Member State nominates their own ECDC NFP for Preparedness and Response. In some cases, ECDC NFPs are the same individuals as those nominated to be WHO International Health Regulation focal points, but the choice depends on the country and there is no specific requirement from the international institutions in this regard. Depending on the organisational structure of the countries, ECDC NFPs for Preparedness and Response could sit in the Ministry of Health or in the National Public Health Institute. The ECDC NFP for Preparedness and Response is responsible for the following: Facilitating links within the health sector and with other sectors for the operational aspects of preparedness and response plans; Ensuring dissemination of information to, and consolidating input from relevant sectors of the administration, including those responsible for other functions related to preparedness and response (surveillance, laboratories, clinics, public health services); Ensuring quick and easy contacts with ECDC for urgent matters; and reviewing Risk Assessments and other documents together with ECDC before they are made public.
