# Supplementary material for: Preparedness and response against diseases with epidemic potential in the European Union: a qualitative case study of Middle East Respiratory Syndrome (MERS) and poliomyelitis in five member states
Source: BMC Health Serv Res. 2018 Jul 6;18:528. doi: 10.1186/s12913-018-3326-0 (PMC6034236; doi:10.1186/s12913-018-3326-0)
Supplement: Supplementary file 1 — Sectors and affiliations of the interviewees in the five participating countries. (PDF 436 kb) [file 12913_2018_3326_MOESM1_ESM.pdf]

Annex 1: Sectors and affiliations of the interviewees in the five participating countries

MERS	Health sector	Non-health sector
**UK**	• Emerging Infections and Zoonoses Infectious Diseases Directorate, Department of Health<br>• Preparedness & Response, Respiratory Diseases Department, Public Health England<br>• News Communications, Public Health England<br>• National Ambulance Resilience Unit<br>• University Tropical and Infectious Disease Unit	• Department for Transportation<br>• Civil Contingencies Secretariat, Cabinet Office<br>• Animal Health and Veterinary Laboratories Agency<br>• Medical Department, Civil Aviation Authority<br>• Border Force Operations<br>• Health and Science Correspondent
**Greece**	• National Focal Point for Preparedness, Ministry of Health<br>• National Institute for Public Health (KEELPNO)<br>• National Health Operations Centre, Ministry of Health<br>• Hellenic Society of Travel Medicine<br>• Infectious Disease Physician<br>• Ambulance Services	• Airport Public Health Authority<br>• Aviation Company Representative<br>• Hellenic Civil Aviation Authority<br>• Health Journalist<br>• Ministry of Rural Development and Food<br>• Ministry of Foreign Affairs, Crisis Management Unit
**Spain**	• Coordinating Centre for Health Alerts and Emergencies, Ministry of Health<br>• Press Office Directorate, Ministry of Health<br>• Foreign Health and Border control, Ministry of Health<br>• Ambulance services in an Autonomous Community<br>• Infectious diseases specialist at University hospital<br>• National Centre for Microbiology<br>• Red Cross	• Animal Health, Ministry of Agriculture<br>• Civil Protection<br>• Ministry of Transport<br>• Ministry of Foreign Affairs<br>• Health Journalist

Poliomyelitis	Health sector	Non-health sector
**Poland**	• Chief Sanitary Inspectorate<br>• Department of Mother and Child, Ministry of Health<br>• Department of Drug Policy and Pharmacy in the Ministry of Health<br>• National Institute of Public Health<br>• National Commission for the Certification of Poliomyelitis Elimination<br>• Head, hospital ward<br>• Regional laboratory<br>• Consultant in Epidemiology<br>• National Focal Point for the International Health Regulations	• Ministry of Foreign Affairs<br>• Ministry of the Interior<br>• Border Guard<br>• Journalist
**Cyprus**	• National Focal Point for Preparedness<br>• Virology laboratory<br>• National Certification Committee for Poliomyelitis Elimination<br>• Government Immunization Centre, Hospital<br>• Central Pharmacy<br>• Infectious Disease physician<br>• Ministry of Health planner<br>• Cyprus Paediatric Society<br>• Red Cross	• Refugee center<br>• Ministry of Foreign Affairs<br>• Ministry of Justice – police/border control<br>• Health journalist
